# Multi-region electricity demand prediction with ensemble deep neural networks

**DOI:** 10.1371/journal.pone.0285456

**Published:** 2023-05-18

**Authors:** Muhammad Irfan, Ahmad Shaf, Tariq Ali, Mariam Zafar, Saifur Rahman, Salim Nasar Faraj Mursal, Faisal AlThobiani, Majid A. Almas, H. M. Attar, Nagi Abdussamiee

**Affiliations:** 1 Electrical Engineering Department, College of Engineering, Najran University, Najran, Saudi Arabia; 2 Department of Computer Science, COMSATS University Islamabad, Sahiwal Campus, Sahiwal, Pakistan; 3 Faculty of Maritime Studies, King Abdualziz University, Jeddah, Saudi Arabia; 4 Centre for Maritime Engineering and Hydrodynamics, Australian Maritime College, University of Tasmania, Launceston, Tasmania, Australia; Jeonbuk National University, KOREA, REPUBLIC OF

## Abstract

Electricity consumption prediction plays a vital role in intelligent energy management systems, and it is essential for electricity power supply companies to have accurate short and long-term energy predictions. In this study, a deep-ensembled neural network was used to anticipate hourly power utilization, providing a clear and effective approach for predicting power consumption. The dataset comprises of 13 files, each representing a different region, and ranges from 2004 to 2018, with two columns for the date, time, year and energy expenditure. The data was normalized using minmax scalar, and a deep ensembled (long short-term memory and recurrent neural network) model was used for energy consumption prediction. This proposed model effectively trains long-term dependencies in sequence order and has been assessed using several statistical metrics, including root mean squared error (RMSE), relative root mean squared error (rRMSE), mean absolute bias error (MABE), coefficient of determination (R^2^), mean bias error (MBE), and mean absolute percentage error (MAPE). Results show that the proposed model performs exceptionally well compared to existing models, indicating its effectiveness in accurately predicting energy consumption.

## Introduction

Energy plays a crucial role in maintaining the social, economic, and environmental sustainability of any nation. Over the last decade, there has been a significant global growth in energy consumption, making energy management vital for nations seeking better economic growth and environmental safety [1]. Predictions regarding energy consumption help in the formulation of energy development and design strategies for energy policies [2]. With energy consumption increasing every passing day in emerging and third world countries, and the anticipated growth in world population to reach 9.5 billion by 2030 [3], there is a prediction of a considerable increase in energy demand. This dramatic increase is expected to double energy demand by 2030, significantly impacting energy consumption [4].

Different machine learning models are being used for the prediction of energy consumption. The method of electric load forecasting involves projection, and in this regard, a Dragonfly algorithm-based quantum support vector regression (SVR) with complete ensemble empirical mode decomposition [4] is utilized. Several hybrid forecasting methods, such as PEM (periodogram estimation method), LSSVR-CCPSO, and SMTs (ship motion time series) that combine PEM, LSSVR (least squares support vector regression) models, and CCPSO (chaotic cloud particle swarm optimization) algorithms have been studied [5]. However, predicting electricity consumption poses several challenges, such as lack of general applicability and difficulties in processing complex data. To predict short-term electricity consumption, several algorithms are combined, and the model’s resilience focuses on the electrical consumption of data. External factors, such as daily, weekly, and seasonal cycles, can also affect its performance. Given that short-term electricity usage data series are nonstationary and nonlinear, there are various frequencies to consider.

The EMD-Fbprophet-LSTM model is utilized to predict short-term electricity consumption, dealing with the complexity and uncertainty of data. LSTM is responsible for forecasting short-term power usage, while Fbprophet is a time series model that decomposes electricity consumption using EMD (Empirical Model Decomposition) for prediction. This new and in-demand model is capable of handling the fluctuating characteristics of electricity demand across different time scales and has a better accuracy rate for short-term electricity consumption. Moreover, it addresses weather-related factors and reduces the forecast rate on customer demand, making it an efficient solution [6].

Load electricity demand forecasting plays a crucial role in intelligent energy management systems [7], where the short-term and long-term loads are considered for the transmission and new infrastructure. The demand for load forecasting is increasing daily due to its significance. Various research papers focus solely on predicting electricity consumption [8], which can be impacted by weather-related factors such as temperature, humidity, and rainfall, as well as socio-economics and population variables [9]. Utilizing general variables can provide better energy consumption results for alternative prediction models. For example, a city power consumption-based forecasting model was developed using the machine learning model Random Forest (RF) to predict power consumption in Agartala Tripura, a city located in India [10]. This model’s validation can help to improve power consumption predictions.

In recent years, there has been a growing interest among researchers in forecasting energy load. Time series data and forecasting models, such as Electrical Power Consumption, have been preferred in most studies. For instance, Nyoni [11] used ARIMA to predict CO2 emission in China from 1977 to 2017 and found that ARIMA (1,2,1) is a suitable method for predicting CO2 emission in China. Similarly, D. E. Rumelhart [12] proposed two models for predicting electricity consumption at UTHM (University Tun Hossein Onn Malaysia). G. Gross [13] suggested ARIMA (4,2,1) as the ideal model for predicting power usage in agriculture. Other methods that have been used for forecasting energy consumption include EDM (Energy Demand Model), ANN (Artificial Neural Network) model, PSO (Particle Swarm Optimization) technique, and GM (Grey Model). A. A. El-Keib [14] utilized EDM for predicting energy consumption in Italian households and industrial states.

The transition from shallow learning to deep learning has been observed in machine learning algorithms. J. Wang [15] proposed a method that uses learning representation with error back-propagation to train an Artificial Neural Network (ANN). In addition, the commonly used machine learning models, such as Decision Tree (DT) and Support Vector Machine (SVM), were used to obtain optimal solutions. With the increase in computing power and improved data accumulation, deep learning algorithms have made complex training easier. In modern times, both deep and shallow learning models have their roles in various fields. The selection of the best machine-learning algorithm for future analysis depends on factors such as data categories, sample size, and model properties. Statistical prediction methods are used for time series forecasting. The prediction of future loads and predictor variables using historical observations is done using load signal and other forecasting tools. Several studies have worked on short-term prediction models, which are for an hour or less and may last up to a week, using linear regression models, multiple regression models, smoothing, and weighted least squares [16, 17].

Additionally, machine learning models such as SVM, ANN, and FIS have also been used for predicting electricity consumption in buildings. SVM has been used to predict the electricity consumption of office buildings based on weather data and historical consumption data [18]. ANN has been used to predict electricity consumption in residential buildings using data such as weather, occupancy, and time of day [19]. FIS has been used to predict the energy consumption of a building based on various factors such as temperature, humidity, and occupancy patterns [17]. These models have shown promising results in accurately predicting electricity consumption in buildings, which can help in optimizing energy usage and reducing energy costs.

J. F. Torres and his team used innovative metering technologies to gather multiple time series datasets and developed a multiple forecasting methodology that utilized RNN with LSTM [20]. Their aim was to achieve a day-ahead prediction of electricity consumption in individual households and industries. They used MSE and MAE performance metrics to evaluate the LSTM model’s performance in predicting time series data on energy consumption. By using LSTM instead of typical univariate and classical methods, they were able to consume a smaller number of computational resources while still achieving accurate predictions.

In [21], Z. Zhang proposed a technique entitled LSTM and CNN to forecast electricity consumption and ensure the smart grid’s suitable operational activities and efficient management. The represented method involves two variates of a sequence, and to obtain the electricity demand forecast, LSTM and ANNS are used to connect a pair of crucial values linked to contextual information, which is then utilized to produce sets. The authors compared the performance of (c,1) LSTM with ARIMA and with sequence to sequence (S2S) LSTM and found that their method offered better accuracy in terms of MAPE and RPSE performance metrics.

M.-W. Li [22] sheds light on the partial electricity demands of the country, keeping an eye on the horizon of 2025 and predicting industrial electricity demand. The authors used a model to predict the electricity demand from 1966 to 2016 and added the following information to make a prediction: the original cost of explanatory variables, the cost of power, its industrial value, and the population of working age. The model shows that the demand for industrial electricity has relatively low long-run elasticities (0.1 and within 0.2-0.3, respectively) for both price and income.

Z.A. khan [23] introduced an efficient method for short-term electricity load forecasting, which is essential for effective energy management. The authors propose a hybrid model that combines the advantages of the autoregressive integrated moving average (ARIMA) and support vector regression (SVR) models. The proposed model is tested using real-world electricity load data obtained from the Pakistan Energy Management Company (PEMCO). The results show that the hybrid model outperforms both the ARIMA and SVR models individually in terms of forecasting accuracy.

In [24], an efficient and effective hybrid model is developed for power generation and consumption forecasting, contributing to energy harvesting by providing valuable prediction data to the concerned renewable energy analysts. The model integrates a convolutional neural network with an echo state network to extract meaningful patterns from historical data and learn temporal features for robust renewable energy generation and consumption forecasting. The output spatiotemporal feature vector is then fed to fully connected layers for final forecasting.

In [25], the proposed ESNCNN model combines an Echo State Network (ESN) and a Convolutional Neural Network (CNN) to learn the nonlinear mapping relationship and extract spatial information from renewable energy data. The authors also use residual connections to avoid the vanishing gradient problem and fully connected layers to enhance and select the optimal features for predicting future energy production.

In [26], the proposed framework combines convolutional neural networks (CNN) with a long short-term memory autoencoder (LSTM-AE) to extract both spatial and temporal features from the electricity consumption data. The CNN is used to extract the spatial information, while the LSTM-AE is used to capture the temporal dependencies. The output of the LSTM-AE is fed into fully connected layers for final forecasting. The proposed framework is evaluated on three real-world datasets and compared with state-of-the-art models using various evaluation metrics.

In [27], the authors proposed a deep feed-forward neural network approach to estimate power usage. They utilized the Apache Spark platform for distributed computing and the H2O data analysis framework. Although different electricity consumption prediction studies are useful depending on specific needs, they are limited by time frames and prediction metrics. In this paper, we propose a deep ensemble neural network for the problem of electric consumption prediction. Our method is compared to various baseline methods that use machine learning algorithms and a multi-sequence LSTM model. Thus, the paper has the following contributions to existing literature:

- Highlighting the importance of electricity consumption prediction in intelligent energy management systems
- Describing the use of a deep-ensembled neural network for predicting power consumption
- Outlining the statistical metrics used to assess the performance of the proposed model, including RMSE, rRMSE, MABE, R^2^, MBE, and MAPE
- Demonstrating the effectiveness of the proposed model in accurately predicting energy consumption, with results showing superior performance compared to existing models

The rest of the paper is organized as follows: Section 2 presents the materials and methods of the proposed work, Section 3 displays the findings of the proposed algorithm on the dataset, Section 4 discusses the purpose and findings of our work, and finally, the paper is concluded in Section 5.

## Materials and methods

### Dataset details

**Fig 1:**
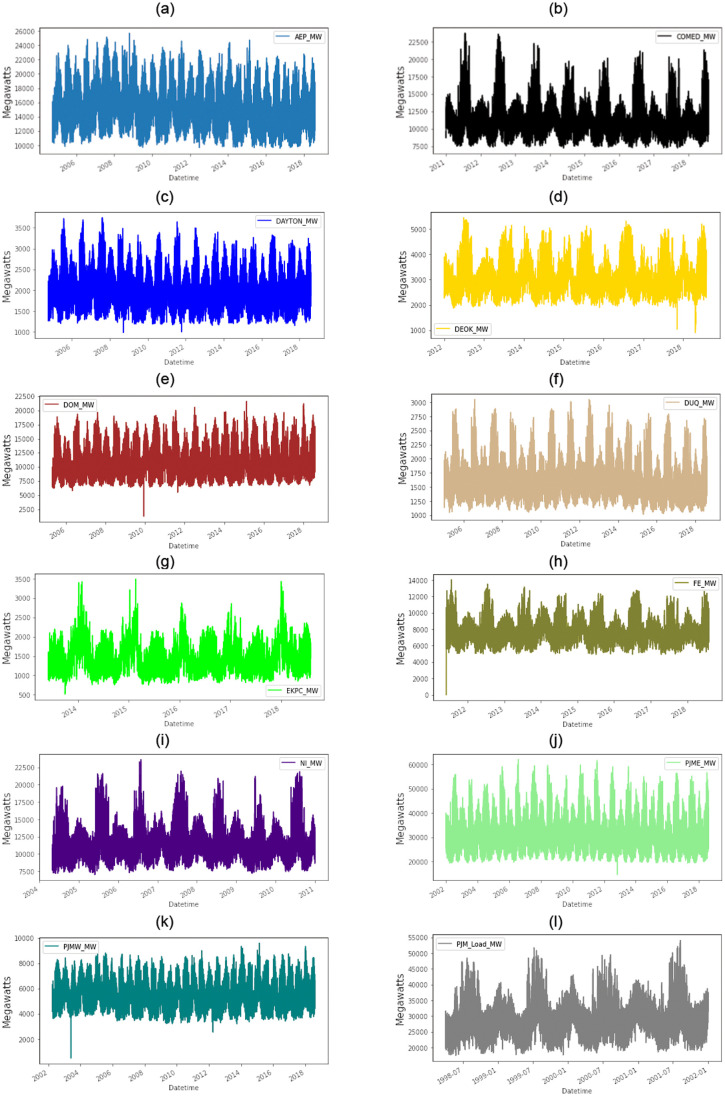
Twelfth regions electricity consumption in Mega Watt.

**Fig 2:**
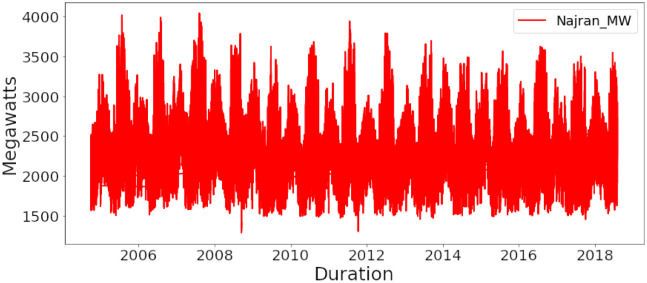
Najran region electricity consumption in Mega Watt.

The dataset contains thirteen files and their graphical representations are presented in Figs 1 and 2. The information mentioned in the files shows per hour utilization of energy. There are two columns in each file. The first column represents the date, time, and year while the second column represents energy expenditure in Megawatts (MW). The first file exhibits the energy consumption rate of the American Electric Power (AEP) region, in which 12,1274 entries in this file show per hour of energy depletion. This file covers the energy dissipation of 14 years. The time series starts on 31st December 2004 at 1:00 A.M showing the energy utilization as 13478 in this particular hour, and ends on 2nd January 2018 at 12:00 A.M, exhibiting the energy consumption rate as 19993.

The second file exhibit the energy consumption of the Commonwealth Edison (ComEd) region. Sixty-six thousand four hundred ninety-eight entries in this file show per hours utilization of energy. This file covers the energy consumption of 7 years. The time series of energy consumption initialized from 31st December 2011 at 1:00 A.M shows the energy utilization as 9970 at this hour. It ends on 2nd January 2018 at 12:00 A.M, exhibiting the energy consumption rate as 12816. The third file exhibit the energy consumption rate of the Dayton Power and Light Company (DAYTON) region. There are 121276 entries in this file that shows per hours depletion of energy. Similar to the AEP region, this file covers the energy dissipation of 15 years. The time series starts on 31st December 2004 at 10:00 A.M showing the energy utilization as 1596 in this hour and it ends on 2nd January 2018 at 12:00 A.M exhibiting the energy consumption rate as 2552. The fourth file exhibit the energy consumption rate of the Duke Energy Ohio/Kentucky (DEOK) region. There are 57740 entries in this file that shows per hours depletion of energy. This file covers the energy dissipation of 6 years. The time series starts on 31st December 2012 at 10:00 A.M showing the energy utilization as 2945 in this hour and it ends on 2nd January 2018 at 12:00 A.M exhibiting the energy consumption rate as 4100.

The fifth file exhibit the energy consumption rate of the Dominion Virginia Power (DOM) region. There are 116190 entries in this file that show per hours depletion of energy. This file covers the energy dissipation of 13 years. The time series starts on 31st December 2005 at 10:00 A.M showing the energy utilization as 9389 in this hour and it ends on 2nd January 2018 at 12:00 A.M exhibiting the energy consumption rate as 17428. The sixth file exhibit the energy consumption rate of the Duquesne Light Co. (DUQ) region. There are 119069 entries in this file that show per hours depletion of energy. This file covers the energy dissipation of 13 years. The time series starts on 31st December 2005 at 10:00 A.M showing the energy utilization as 1458 in this hour and it ends on 2nd January 2018 at 12:00 A.M exhibiting the energy consumption rate as 1721. The seventh file exhibit the energy consumption rate of the East Kentucky Power Cooperative (EKPC) region. There are 45335 entries in this file that shows per hours depletion of energy. This file covers the energy dissipation of 5 years. the time series starts on 31st December 2013 at 10:00 A.M showing the energy utilization as 1861 at this hour and it ends on 2nd January 2018 at 12:00 A.M exhibiting the energy consumption rate as 2846.

The eighth file exhibit the energy consumption rate of the FirstEnergy (FE) region. There are 62875 entries in this file that show per hours depletion of energy. This file covers the energy dissipation of 7 years. the time series starts on 31st December 2011 at 10:00 A.M showing the energy utilization as 6222 at this hour and it ends on 2nd January 2018 at 12:00 A.M exhibiting the energy consumption rate as 8393. The ninth file exhibit the energy consumption rate of the Northern Illinois Hub (NI) region. There are 58451 entries in this file that show per hours depletion of energy. This file covers the energy dissipation of 6 years. The time series starts on 31st December 2004 at 10:00 A.M showing the energy utilization as 9810 in this hour and it ends on 2nd January 2010 at 12:00 A.M exhibiting the energy consumption rate as 12223.

The tenth file exhibit the energy consumption rate of the PJM East Region: 2001-2018 (PJME) region. There are 145367 entries in this file that show per hours depletion of energy. This file covers the energy dissipation of 16 years. the time series starts on 31st December 2002 at 10:00 A.M showing the energy utilization as 26498 in this hour and it ends on 2nd January 2018 at 12:00 A.M exhibiting the energy consumption rate as 38608. The eleventh file exhibit the energy consumption rate of the PJM West Region: 2001-2018 (PJMW) region. There are 143207 entries in this file that show per hours depletion of energy. This file covers the energy dissipation of 14 years. The time series starts on 31st December 2002 at 1:00 A.M showing the energy utilization as 5077 in this hour and it ends on 2nd January 2010 at 12:00 A.M exhibiting the energy consumption rate as 7691.

The twelfth file exhibit the energy consumption rate of the PJM Load Combined: 1998-2001 (PJM_Load) Load region. There are 32897 entries in this file that show per hours depletion of energy. This file covers the energy dissipation for 3 years. The time series starts on 31st December 1998 at 10:00 A.M showing the energy utilization as 28309 in this hour and it ends on 2nd January 2001 at 12:00 A.M exhibiting the energy consumption rate as 29506. Najran is also added to the list for prediction of energy consumption as shown in Fig 2. Najran dataset consists of the following sectors: Governmental, agricultural, residential, commercial, industrial, and others. The dataset availability was year-wise from 2005 to 2018. Energy consumption of different sectors in 2005 have been shown in Table 1.

**Table 1:** Energy consumption of Najran region in 2005.

Sector	Energy Consumption (MW)
Governmental	122164
Agricultural	52674
Residential	8320650
Commercial	867081
Industrial	105349
Others	442594

The data shows that total energy consumption in 2005 was 11009989 MW. Similarly, the energy consumption in 2018 was 28679063 MW and the sum of energy consumption in 14 years (i.e., 2005-2018) was 283895206 MW. The total sum is divided over the period of 14 years to get hour-based energy consumption values. There are 112516 entries in this file that show per hours depletion of energy. The time series starts on 1st January 2005 at 00:00 A.M showing the energy utilization as 2096 MW in this hour and it ends on 31st December 2018 at 23:00 P.M, exhibiting the energy consumption rate as 3052 MW. The hourly consumption values are being used in the neural network model for the prediction of energy utilization.

### System architecture

**Fig 3:**
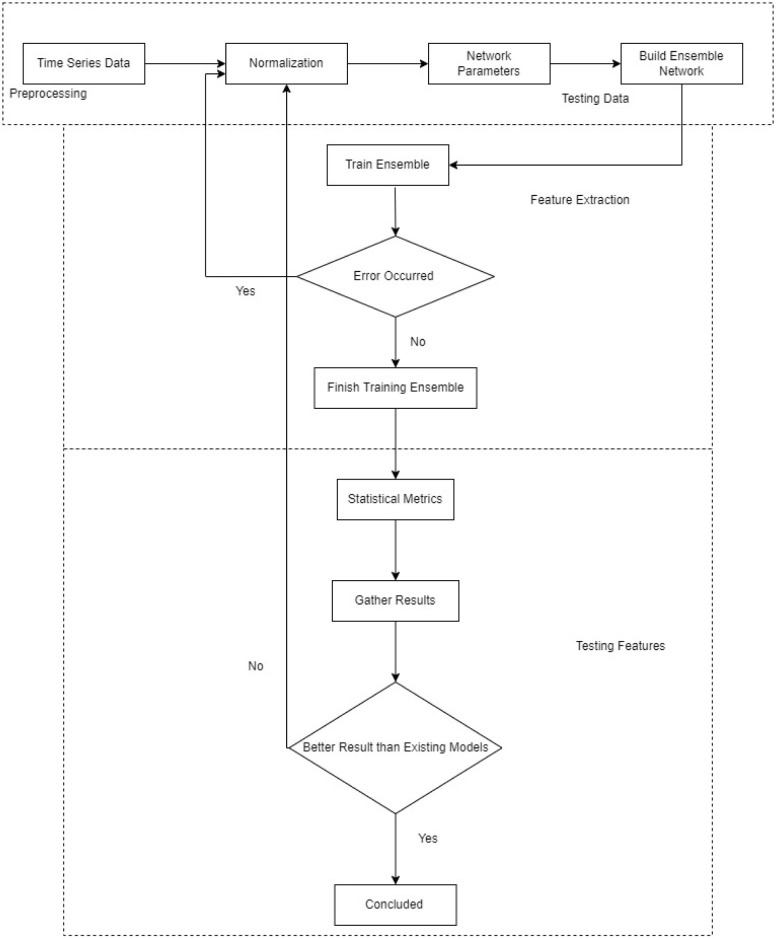
Flow chart of proposed methodology.

The proposed methodology is depicted in Fig 3. It begins by choosing the time series data to be normalized. The network parameters are then selected. The network parameters serve as the foundation for the ensemble model. Next, training for the ensemble model is initialized. The error’s occurrence is then examined. The ensemble training would be over if the error happened. Additionally, the procedure will be repeated if no errors have occurred. When the statistical measurements are examined in the third phase, feature fusion takes place. The ensemble model is developed and evaluated against previous research. The process will be finished if the findings are satisfactory; the normalization step will be repeated again. Furthermore, the detailed description of the system architecure has been explained in the following sections: Dataset Preprocessing, Proposed Deep Ensemble Learning, Recurrent Neural Network and Long Short Term Memory.

#### Dataset preprocessing

The performance of machine learning algorithms tends to increase with the scaling of numerical input to a standard range. Two types of algorithms are included. The first one is used to measure the distance such as KNN (K-nearest neighbor) and the second one is helpful for weighting sum of the input such as linear regression. Normalization and standardization are the two most widely used methods for the scaling of numeric data before modeling. Each variable is scaled separately to a scope of 0-1, which is the breaking point for movable information where most exactness is in Standardization.

Accordingly, for every variable, the size and measure of data removed from the area might contrast. It’s feasible for input factors to have unmistakable scales due to their various units (like feet, kilometers, and hours). The intricacy of the test being demonstrated might be exacerbated by contrasts in scales across input factors. A model might learn huge weight values because of having enormous info values, like a scattering of tens or many units. Enormous weight values show that the model is temperamental, and that implies that it might perform inadequately during learning and be delicate to enter values, prompting a higher speculation error. Scikit library helps in achieving both data normalization and standardization. By rescaling the data from the previous range to a new range between 0 and 1, normalization brings all values into the new range. one must be aware of or be able to precisely calculate the maximum and minimum observable values in order to perform normalization. This is how a value is normalized:

$$\begin{array}{c} y=\frac{\text{max}-\text{min}}{\text{max}-\text{min}} \end{array}$$ (1)

Here, output data is scaled in form of Y. Data input by X. column’s minimal value is shown as min. a column’s maximum value is shown as max. If there are local variables in the dataset, MinMax Scaler scales all of the observed data in the range [-1, 1] otherwise. All of the successive thing sets in the limited reach [0, 0.005] are packed by this scaling. Because of the effect of the irregularities during the calculation of actual mean and standard deviation, Standard Scaler doesn’t guarantee adjusted highlight scales in that frame of mind of exceptions. The range of the characteristic qualities consequently decreases. The following are some best practices for using the MinMax Scaler and other scaling techniques: 1. Adapt the scaler by using the classification model provided. Regarding normalization, this means that minimal and maximum accessible supervised learning will be used to calculate values. To do this, use the fit() function. 2. The scale can be applied to the normalization of data. This elaborates that you can train your algorithm using normalized data. Calling the transform() method accomplishes this. 3. The scale can also be used for forwarding data. This implies that you can prepare fresh data in the future for your predictions.

#### Proposed deep ensemble learning

Different algorithms are used to predict the future energy consumption. However, a single model accuracy may not be considered adequate for the given dataset because every algorithm has limitations, and it is not easy to cope with challenges with just one choice. For this reason, different algorithms are combined to boost the results. Gathering strategies is a machine learning procedure that unites a couple of base models to make one ideal best model. Ensemble learning is a general way to deal with machine learning that looks for better prescient execution by consolidating the prediction from different models.

Although there are an apparently limitless number of ensembles that can be produced for the prescient displaying issue, three strategies dominate the field of ensemble learning. The three principal classes of ensemble learning strategies are boosting, stacking, and bagging. It is essential to both have a detailed comprehension of every technique and to think of them as on predictive displaying project. The objective of any ML issue is to determine solitary methods that will foresee the needed result. Instead of making one model and trusting this model is an excellent precise indicator, we can make ensemble models consider a bunch of methods and normalize those models to deliver one last mode.

#### Recurrent neural network

**Fig 4:**
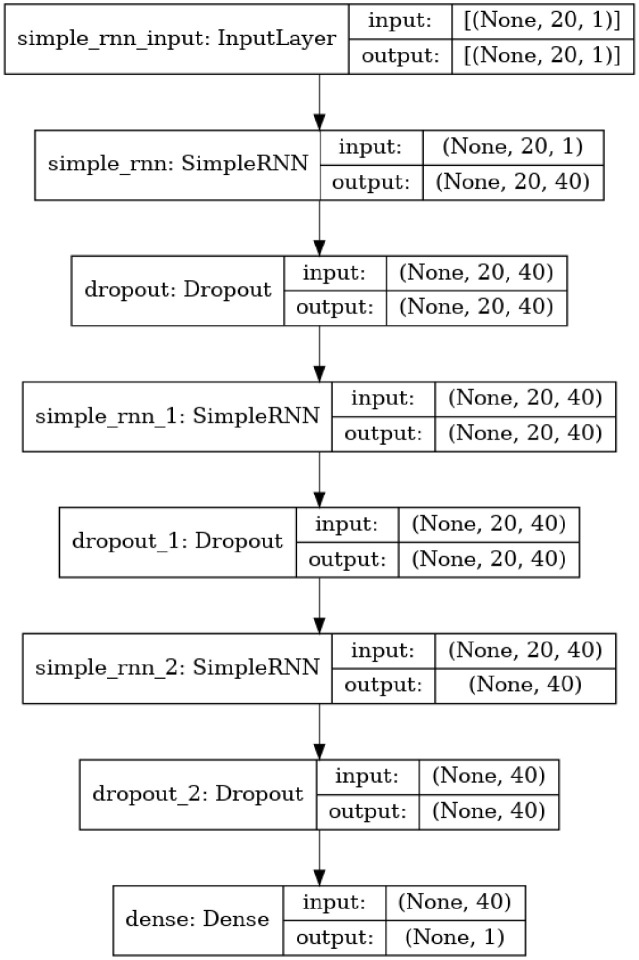
RNN architecture in proposed work.

A specific type of neural network is recurrent neural networks (RNNs) where the calculations at each step are informed by the outcomes of the previous stage. Inputs and outputs are present in conventional neural networks that are independent of one another. The hidden layer saves data about a succession, and it is the essential and generally critical quality of RNNs as shown in Fig 4. To prepare the dataset utilizing RNN, the info is given to the organization in a solitary time step. The framework’s present status is then resolved utilizing the arrangement of current information and the past state. The ongoing time becomes time-1 for the ensuing time step. One can make as many time strides in the past as needs be and blend the information from every one of the old states, contingent upon the issue. After each time step has finished, the result is determined utilizing the last present status. When the result is diverged from the objective result, which is the genuine result, the error is then delivered. To refresh the loads, the network (RNN) is prepared after the error is backpropagated to it [28].

Using sequences to learn, RNN is a deep learning model. The past output is added to the current input by RNN in a recursive manner. RNN’s current output uses the past output to learn from the previous sequence. The sequence is given to the current input as second input. As a result, the previous sequences would have an impact on the current output. The past sequences combine to continue the past outcomes. Each data point at time t occurs in reference to the past data in a dataset that flows sequentially. RNN has been used in machine learning to extract the fundamental pattern and meaning from such consecutive information [29]. In specific, a solitary information is applied to a tapped-delay-line memory of s units in an engineering of info yield repetitive model in view of the MLP, and a solitary result is taken care of once more into the information by means of one more tapped-delay-line memory of t units. This two-tapped delay-line memory provides data to the MLP’s input layer. In other words, the output precedes the input by one temporal unit. u (n) stands for the current value of the model info, and y (n+1) represents the equivalent value of the model result. To reflect exogenous information sources that came from outside the network, the sign vector provided to the information layer of the MLP hence comprises of an information window comprised of over a wide span of time upsides of the plant inputs. This is because of the way that the model results are relapsed on their postponed values. The nonlinear auto backward with exogenous information sources (NARX) model is the name of this repetitive network [30].

#### Long short term memory

Predictive modeling challenges involving time series prediction are complex. Time series, in contrast to regression predictive modeling, also increases complexity by introducing a relationship between the input variables’ sequences. Recurrent neural networks are a potent class of neural networks built to manage sequence dependence. Because very large designs may be taught, the Long Short-Term Memory network, also referred to as the LSTM network, is a type of RNN used in machine learning. Using backpropagation across time, it was trained. As a result, it is used to build substantial recurrent networks, which can then be utilized to tackle challenging sequence issues in machine learning and produce cutting-edge outcomes. Memory blocks in LSTM networks are connected by layers as opposed to neurons [31].

**Fig 5:**
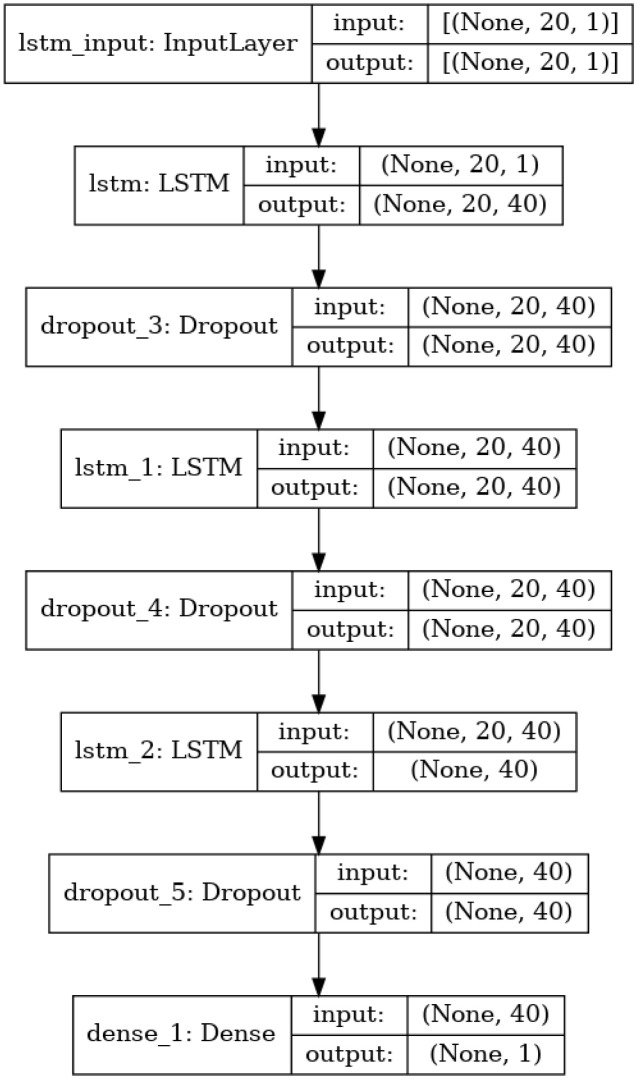
LSTM architecture in proposed work.

It is easy to understand the temporal relation between sequences with the LSTM functionality as depicted in Fig 5. Exploding and vanishing gradient issues are resolved by its internal memory unit and gate mechanism, which are issues with typical RNN training. Input gate, output gate, forget gate, and cell status are the four significant units that make up the LSTM model’s internal structure. These three gates are responsible for the Maintenance and updating of information in cell status. Following Eqs (2)–(7) show the computational process

$$\begin{array}{c} qt=\sigma (pq[pt-1,yt]+bq) \end{array}$$ (2)

$$\begin{array}{c} rt=\sigma (pw[pt-1,yt]+br) \end{array}$$ (3)

$$\begin{array}{c} st=\sigma (pe[pt-1,yt]+bs) \end{array}$$ (4)

$$\begin{array}{c} wt=\text{tanh}(pr[pt-1,yt]+bw) \end{array}$$ (5)

$$\begin{array}{c} ut=ft*ut-1+rt*wt \end{array}$$ (6)

$$\begin{array}{c} vt=ot*\text{tanh}(ut) \end{array}$$ (7)

*σ* is called the sigmoid activation function. The forget, input, and output gates values are denoted by the notations qt, rt, and st, respectively. The memory cell is referred to as ut and updating and activating the present cell status is at. The output vector result at time t is represented by vectors vt and yt, respectively. pq,r,w,s and bq,r,w,s are the bias vectors and the weights matrices, respectively [32, 33]. Network Parameters of LSTM: Trainable weights also known as trainable parameters are used to identify the complexity of the network. The layers of the network such as input layer, output layer and hidden layer exemplify the trainable weights into the structure and internal connections of LSTM. The total number of tranable weights can be calculated as:

$$\begin{array}{c} TWS=4iL+4LL+4L+oL+o \end{array}$$ (8)

Where, TWS = Number of trainable weights, i = inputs, o = output, L = LSTM cells in the hidden layers. The difference between the poor and good performance means the selection of finest parameters for the neural network architecture.

## Results

This paper proposed a method to predict energy consumption of thirteen regions. The method utilized the publicly available dataset of twelve region on Kaggle and one region (Najran) data from https://data.gov.sa/Data/en/dataset. Following libraries have been used for energy prediction: Numpy array, pandas, tensorflow, matplotlib, sequential model, from keras layers: dense, RNN, LSTM, and dropout.

### Model evaluation

The following Regression Metrics: root mean squared error (RMSE), relative root mean squared error (RMSE), mean absolute bias error (MABE), coefficient of determination (R^2^), mean bias error (MBE) and mean absolute percentage error (MAPE) help in evaluating machine Learning models. Statistical equations of the regression metrics have been shown in Eqs (9)–(14) where uw represents the acutal value, nw is the predicted value, and K represent number of observations.

$$\begin{array}{c} MBE=\frac{1}{k}\sum_{w=1}^{k}{\left(u_{w}-n_{w}\right)}^{2} \end{array}$$ (9)

$$\begin{array}{c} RMSE=\sqrt{\frac{1}{k}\sum_{w=1}^{k}{\left(u_{w}-n_{w}\right)}^{2}} \end{array}$$ (10)

$$\begin{array}{c} MABE=\frac{1}{k}\sum_{w=1}^{k}\left|u_{w}-n_{w}\right| \end{array}$$ (11)

$$\begin{array}{c} R^{2}=1-\frac{\sum_{w=1}^{k}{\left(u_{w}-n_{w}\right)}^{2}}{\sum_{w=1}^{k}{\left(u_{w}-\bar{n_{w}}\right)}^{2}} \end{array}$$ (12)

$$\begin{array}{c} MAPE=\frac{1}{k}\sum_{w=1}^{k}|\frac{u_{w}-n_{w}}{u_{w}}| \end{array}$$ (13)

$$\begin{array}{c} rRMSE=\sqrt{\frac{RMSE}{\sum_{w=1}^{k}n_{w}^{2}}*100} \end{array}$$ (14)

### Energy prediction of proposed model

**Fig 6:**
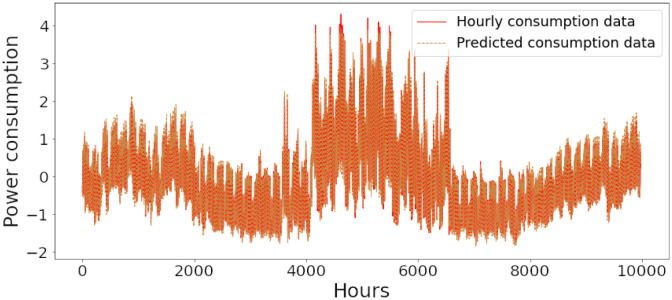
Energy consumption prediction of NI.

**Fig 7:**
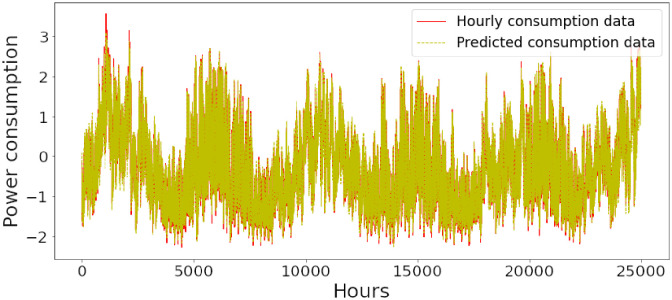
Energy consumption prediction of AEP.

**Fig 8:**
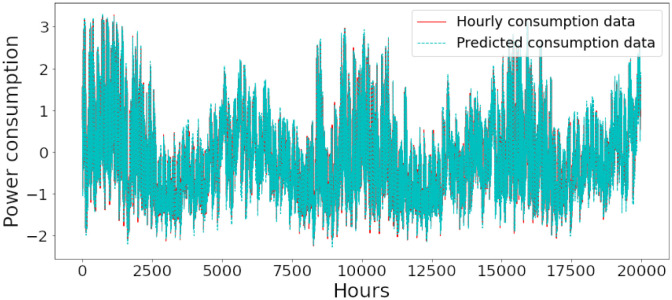
Energy consumption prediction of DAYTON.

**Fig 9:**
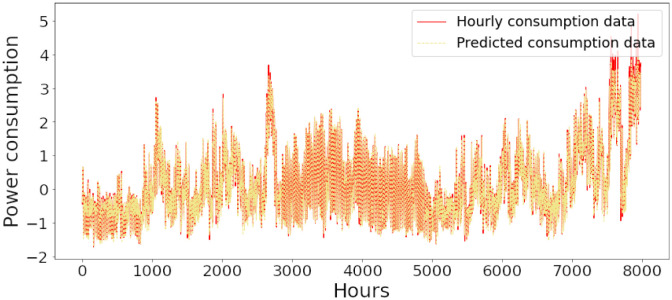
Energy consumption prediction of EKPC.

**Fig 10:**
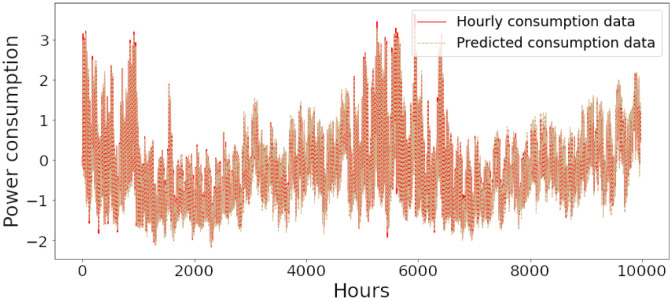
Energy consumption prediction of FE.

**Fig 11:**
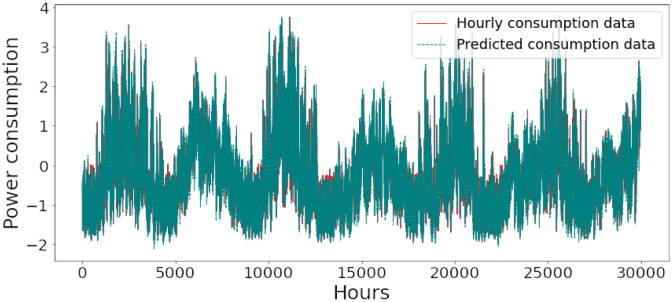
Energy consumption prediction of PJME.

**Fig 12:**
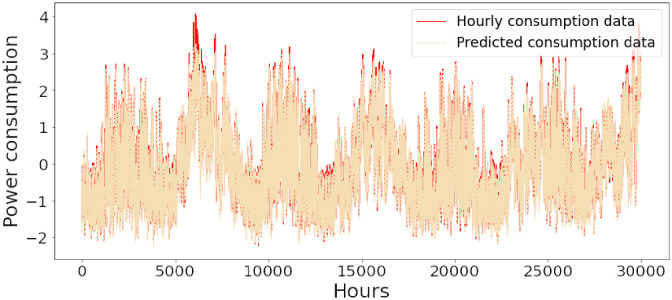
Energy consumption prediction of PJMW.

**Fig 13:**
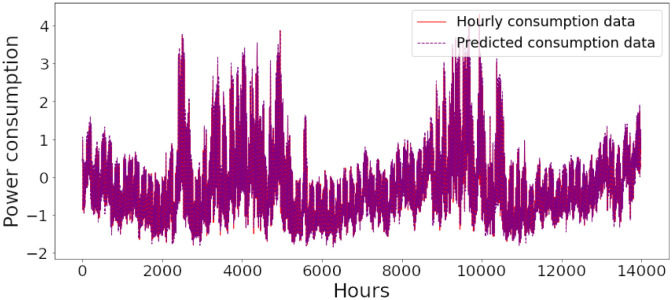
Energy consumption prediction of COMED.

**Fig 14:**
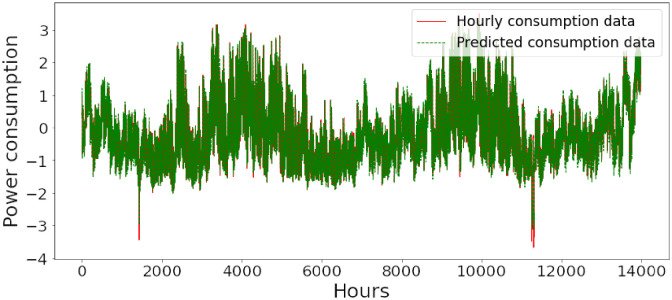
Energy consumption prediction of DEOK.

**Fig 15:**
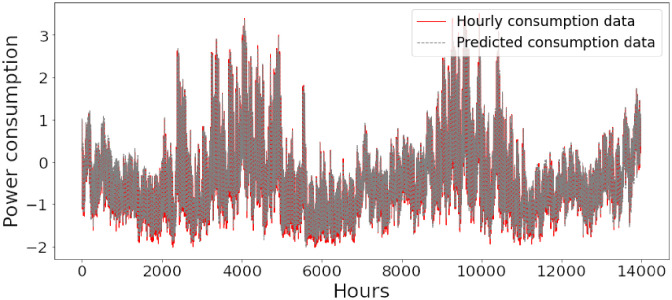
Energy consumption prediction of DUQ.

**Fig 16:**
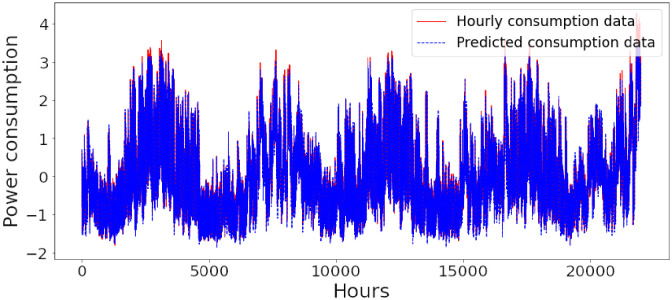
Energy consumption prediction of DOM.

**Fig 17:**
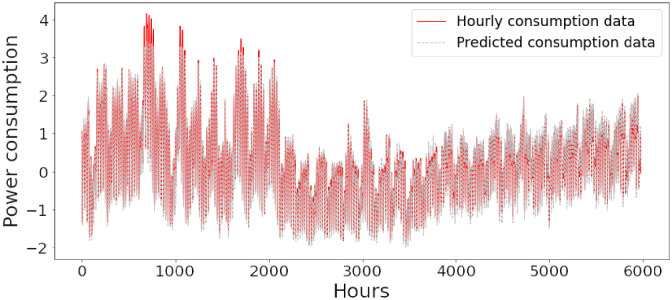
Energy consumption prediction of PJM_Load.

**Fig 18:**
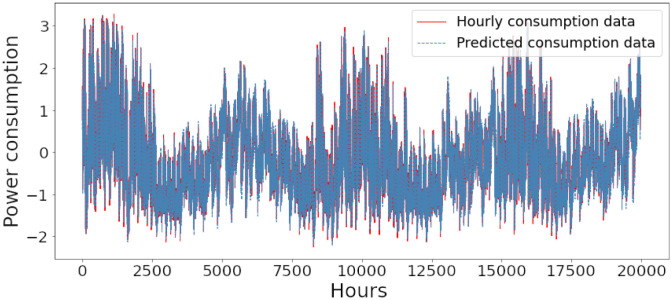
Energy consumption prediction of Najran.

Figs 6–18 represent the proposed model’s absolute and predicted values on thirteen regions’ energy consumption. In the figures, the solid red line shows the actual value of all regions. In contrast, the dotted line (different colors for each region) exhibits the predicted value of the proposed model. The X-axis holds several testing hours (TH) while the Y-axis sets out the normalized value of hourly energy consumption of all regions. Fig 6 shows the energy consumption of the NI region, where 10,000 hours are used as a testing unit to predict the performance of the proposed model. Fig 7 represents the energy expenditure of the AEP region in which 25,000 hours are tested to analyze the regression metrics of the proposed model. Fig 8 exhibits the power utilization of the DAYTON region, having 20,000 testing hours for the validation of predicted results. Fig 9 displays the electricity consumption of the EKPC region where 8000 testing hours are mapped on the predicted value of the proposed model.

Fig 10 explains the energy consumption of the FE region in which 10,000 hours have been used to verify obtained results. Fig 11 discusses the energy consumption for the PJME region. Thirty thousand testing hours are taken to check the performance of the proposed model. The energy consumption for the PJMW region is shown in Fig 12, in which 30,000 testing hours are captured to validate the proposed model’s performance. Fig 13 discusses the energy consumption for the COMED region that addresses the prediction of 14,000 testing hours. The energy consumption for the DEOK region is shown in Fig 14, where the projection of actual and predicted values of 14,000 hours are displayed. The energy usage for the DUQ region is covered in Fig 15. For DUQ, 14,000 hours are used for testing the proposed model. Fig 16 displays the DOM region’s energy usage. It depicts the consumption of 22,000 hours to verify the accuracy of the proposed model. Fig 17 shows how much energy is used in the PJML area. In this region, 6000 testing hours are considered for analyzing system performance. In Fig 18, the finding for the Najran region is displayed. For Najran, 20,000 testing hours are utilized to analyze the proposed model’s prediction accuracy.

In terms of R^2^ values, the proposed model achieves higher in the PJME region and lower in the PJM_Load region. In the case of MAPE values, the proposed model reaches a higher error value in PJM_Load and lower in the DAYTON region. In terms of MBE values, the proposed model attains higher in the EKPC region and lower in the PJME region. Regarding RMSE values, the proposed model is secured higher in the EKPC region and lower in the COMED region. Regarding rRMSE values, the proposed model obtains higher in PJM_Load region and lower in the PJME region. In terms of MABE values, the proposed model gains higher in PJME_Load region and lower in the PJME region.

The proposed study analyzes the data of 13 regions and exhibits the values of R^2^, MAPE, MBE, RMSE, rRMSE, and MABE as shown in Table 2. RNN and LSTM models were ensembled to get the results of these values. The average accuracy rate for R^2^ is 0.9731. while MAPE gives an average of 1.9238, MBE offers 0.0281, RMSE gives 0.1658, rRMSE exhibits 0.0326, and MABE gives a value of 0.1809. A comparison experiment is designed between existing research studies and proposed work, showing that the accuracy rate is higher for a given energy consumption dataset. The main focus is to come by an improved outcome at the results. As Table 3 shows [34], has excellent accuracy rates, but it only offers the potential gains of MAPE and RMSE.

**Table 2:** Regression analysis of proposed models.

Regions	TH	R^2^	MAPE	MBE	RMSE	rRMSE	MABE
AEP	25000	0.9742	1.8641	0.024	0.155	0.0253	0.0986
COMED	14000	0.9761	2.187	0.0224	0.1497	0.0337	0.0943
DAYTON	20000	0.975	1.6523	0.0246	0.1568	0.0283	0.0985
DEOK	14000	0.9722	2.4265	0.0297	0.1723	0.034	0.1118
DOM	22000	0.9731	1.7218	0.029	0.1704	0.0266	0.1045
DUQ	14000	0.9792	1.8522	0.0227	0.1506	0.0242	0.0993
EKPC	8000	0.9474	1.8535	0.0587	0.2424	0.0517	0.1485
FE	10000	0.9762	1.8157	0.0229	0.1512	0.0397	0.997
NI	10000	0.9791	1.953	0.0228	0.1509	0.0372	0.0957
PJME	30000	0.9814	1.8231	0.0191	0.1383	0.0211	0.0874
PJMW	30000	0.9746	1.6844	0.0281	0.1677	0.0225	0.1066
PJM_LOAD	6000	0.9689	2.4515	0.0356	0.1888	0.0512	0.1289
NAJRAN	20000	0.973117	1.940425	0.027969	0.166175	0.03295833	0.180925

**Table 3:** Comparison of regression analysis.

References	R^2^	MAPE	MBE	RMSE	rRMSE	MABE
[34]	-	14.6	-	444.5071	-	-
[35]	-	35.53	-	-	-	-
[36]	0.3806	-	-	0.6328	-	-
[37]	0.985	2.889	-	4.127		-
[38]	-	43.97	-	0.5558	-	-
Proposed Model	0.9731	1.9238	0.0281	0.1658	0.0326	0.1809

Similarly, in [35], inspects the potential gains of MAPE. It‘s values help to get accuracy rates, yet it gives no direction about other indispensable factors. In [36], shows the potential gains of R^2^ and RMSE [37], looks at R^2^, MAPE, and RMSE, while [38] shows MAPE and RMSE. This vast number of studies has proposed a savvy thought for deciding power use for the future, yet the fundamental drawback is the shortfall of critical components and recipes.

## Discussion

The review proposed an ensemble model for gauging power utilization in the hourly time frame. The proposed model plans to convey the best outcomes in forecasting energy consumption. The ensemble approach utilized in this research makes the precision rate higher. A solitary calculation can be utilized to foresee the exact pace of the given dataset, yet it isn’t dependable in some cases. Gathering at least two models can offer far and away superior outcomes and increment the general accuracy rate. The study makes a grouping of algorithms (RNN and LSTM), the initial load might vary however the plan for these models is kept something very similar.

The methodology is applied to the given dataset to make veritable determining. The field of group learning is generally around mulled over and there are various minor takeoffs from this direct point. RNN is utilized in this concentrate because of its outperformance in estimating the utilization of energy. RNN is the most ideal decision for dealing with a gathering of comparable data. RNNs apply loads to the current and the previous data. Moreover, a tedious neural network will similarly change the heaps for both the tendency to dive and backpropagation through time. The given dataset contains the previous data of quite a while (various periods of time for every region). This data is useful for RNN to make predictions about the impending years. Alongside RNN, LSTM is utilized in this research to make the outcomes more dependable and bona fide.

The mix of LSTM and RNN guarantees a higher exactness rate for gauging future utilization. LSTM is a momentous sort of RNN, which shows excellent execution on a tremendous assortment of issues. LSTM networks track down significant applications in the going with areas: Language showing, Machine translation, penmanship affirmation, Picture captioning, Picture age using thought models, and Question tending. It is different RNNs that are great for learning extended-length conditions, particularly in get-together deciding issues. LSTM has discussion affiliations, i.e., it is ready for dealing with the whole movement of data, other than single server farms like pictures.

A correlation exploration is held between existing research and proposed work which clearly shows the precision rate is higher for a given dataset of power consumption. The research analyzes precision pace of R^2^, MAPE, MBE, RMSE, rRmse, and MABE. The inspiration is to get a better result at the outcomes. As the table shows [34], has impressive precision rates yet it just offers the upsides of MAPE and RMSE. Anticipating the precision of one more significant calculation in its research is troublesome.

Likewise, in [35] authors examine the upsides of MAPE only. MAPE values are useful to get precision rates, yet it provides no guidance about other vital variables. Some other studies are likewise audited for a similar reason, for example [36], shows the upsides of R^2^ and RMSE [37], examines R^2^, MAPE, and RMSE, while [38] shows MAPE and RMSE. This large number of studies have proposed a smart thought for determining power utilization for the future, yet the main disadvantage is the absence of significant elements and recipes. This prompts the point that the results show that both LSTM and RNN offer the best results with higher accuracy and the blunder rate is extremely low. Moreover, LSTM and RNN furthermore assisted in choosing most minor blunders conversely, with various models, for instance, ARIMA and SARIMA which didn’t offer satisfactory results.

## Conclusion

This study uses LSTM and RNN because of their capacity to manage the consecutive information and extraordinary attributes of keeping up with transient connections in long haul. The proposed model aides in deciding different forecasting models for energy consumption. A comparison experiment is held between previously existing studies and proposed work which obviously shows the accuracy rate is higher for given dataset of power consumption. The dataset is partitioned into 13 distinct regions and every locale shows the hourly energy consumption for a particular time frame length. The model is prepared to manage the dataset in time series grouping. When the model is prepared and optimal values are determined, the best LSTM and RNN network is applied to the information to foresee hourly utilization of energy. The forecasting is made for the next years in the dataset. The outcomes show that ensembled LSTM and RNN offer the best outcomes with higher precision and the error rate is very low. Besides, LSTM and RNN additionally helped in deciding most minor errors as contrast with different models, for example, ARIMA and SARIMA which didn’t offer fulfilled results. The future motivation is to create hybrid models with much higher precision and higher paces. The work can be additionally improved by adding records of a few other helpful variables to cover more urgent perspectives. The proposed stud provides a clear guidance to forecasting energy consumption by outflanking different models and can be utilized as a most ideal decision for future expectations.

## References

[1] Fiot JB, Dinuzzo F. Electricity demand forecasting by multi-task learning. IEEE Transactions on Smart Grid. 2016 May 10;9(2):544–51. doi: 10.1109/TSG.2016.2555788

[2] Nizar AH, Zhao JH, Dong ZY. Customer information system data pre-processing with feature selection techniques for non-technical losses prediction in an electricity market. In2006 International Conference on Power System Technology 2006 Oct 22 (pp. 1–7). IEEE.

[3] Viegas JL, Esteves PR, Melicio R, Mendes VM, Vieira SM. Solutions for detection of non-technical losses in the electricity grid: A review. Renewable and Sustainable Energy Reviews. 2017 Dec 1;80:1256–68. doi: 10.1016/j.rser.2017.05.193

[4] Meng Z, Sun H, Wang X. Forecasting energy consumption based on SVR and Markov model: A case study of China. Frontiers in Environmental Science. 2022:363.

[5] Suganthi L, Samuel AA. Energy models for demand forecasting—A review. Renewable and sustainable energy reviews. 2012 Feb 1;16(2):1223–40. doi: 10.1016/j.rser.2011.08.014

[6] Sotomane C, Asker L, Massingue V. ICT for automated forecasting of electrical power consumption: A case study in Maputo. In2011 IST-Africa Conference Proceedings 2011 May 11 (pp. 1–8). IEEE.

[7] Mukherjee A, Kundu PK, Das A. Power system fault identification and localization using multiple linear regression of principal component distance indices. Int. J. Appl. Power Eng. 2020 Aug;9(2):113–26.

[8] Bolandnazar E, Rohani A, Taki M. Energy consumption forecasting in agriculture by artificial intelligence and mathematical models. Energy Sources, Part A: Recovery, Utilization, and Environmental Effects. 2020 Jul 2;42(13):1618–32. doi: 10.1080/15567036.2019.1604872

[9] Aurna NF, Rubel MT, Siddiqui TA, Karim T, Saika S, Arifeen MM, et al. Time series analysis of electric energy consumption using autoregressive integrated moving average model and Holt Winters model. TELKOMNIKA (Telecommunication Computing Electronics and Control). 2021 Jun 1;19(3):991–1000. doi: 10.12928/telkomnika.v19i3.15303

[10] Farajian L, Moghaddasi R, Hosseini S. Agricultural energy demand modeling in Iran: Approaching to a more sustainable situation. Energy Reports. 2018 Nov 1;4:260–5. doi: 10.1016/j.egyr.2018.03.002

[11] Gori F, Takanen C. Forecast of energy consumption of industry and household & services in Italy. International Journal of Heat and Technology. 2004;22(2):115–21.

[12] Rumelhart DE, Hinton GE, Williams RJ. Learning representations by back-propagating errors. nature. 1986 Oct 9;323(6088):533–6. doi: 10.1038/323533a0

[13] Gross G, Galiana FD. Short-term load forecasting. Proceedings of the IEEE. 1987 Dec;75(12):1558–73. doi: 10.1109/PROC.1987.13927

[14] El-Keib AA, Ma X, Ma H. Advancement of statistical based modeling techniques for short-term load forecasting. Electric Power Systems Research. 1995 Oct 1;35(1):51–8. doi: 10.1016/0378-7796(95)00987-6

[15] Wang J, Li L, Niu D, Tan Z. An annual load forecasting model based on support vector regression with differential evolution algorithm. Applied Energy. 2012 Jun 1;94:65–70. doi: 10.1016/j.apenergy.2012.01.010

[16] Khayatian F, Sarto L. Application of neural networks for evaluating energy performance certificates of residential buildings. Energy and Buildings. 2016 Aug 1;125:45–54. doi: 10.1016/j.enbuild.2016.04.067

[17] Ascione F, Bianco N, De Stasio C, Mauro GM, Vanoli GP. Artificial neural networks to predict energy performance and retrofit scenarios for any member of a building category: A novel approach. Energy. 2017 Jan 1;118:999–1017. doi: 10.1016/j.energy.2016.10.126

[18] Alonso AM, Nogales FJ, Ruiz C. A single scalable LSTM model for short-term forecasting of massive electricity time series. Energies. 2020 Oct 13;13(20):5328. doi: 10.3390/en13205328

[19] Kim M, Choi W, Jeon Y, Liu L. A hybrid neural network model for power demand forecasting. Energies. 2019 Mar 10;12(5):931. doi: 10.3390/en12050931

[20] Surono S, Goh KW, Onn CW, Marestiani F. Developing an optimized recurrent neural network model for air quality prediction using K-means clustering and PCA dimension reduction. International Journal of Innovative Research and Scientific Studies. 2023 Mar 21;6(2):330–43. doi: 10.53894/ijirss.v6i2.1427

[21] Zhang Z, Hong WC. Electric load forecasting by complete ensemble empirical mode decomposition adaptive noise and support vector regression with quantum-based dragonfly algorithm. Nonlinear dynamics. 2019 Oct;98:1107–36. doi: 10.1007/s11071-019-05252-7

[22] Li MW, Geng J, Hong WC, Zhang LD. Periodogram estimation based on LSSVR-CCPSO compensation for forecasting ship motion. Nonlinear Dynamics. 2019 Sep;97:2579–94. doi: 10.1007/s11071-019-05149-5

[23] Khan ZA, Ullah A, Haq IU, Hamdy M, Maurod GM, Muhammad K, et al. Efficient short-term electricity load forecasting for effective energy management. Sustainable Energy Technologies and Assessments. 2022 Oct 1;53:102337. doi: 10.1016/j.seta.2022.102337

[24] Khan ZA, Hussain T, Baik SW. Boosting energy harvesting via deep learning-based renewable power generation prediction. Journal of King Saud University-Science. 2022 Apr 1;34(3):101815. doi: 10.1016/j.jksus.2021.101815

[25] Khan ZA, Hussain T, Haq IU, Ullah FU, Baik SW. Towards efficient and effective renewable energy prediction via deep learning. Energy Reports. 2022 Nov 1;8:10230–43. doi: 10.1016/j.egyr.2022.08.009

[26] Khan ZA, Hussain T, Ullah A, Rho S, Lee M, Baik SW. Towards efficient electricity forecasting in residential and commercial buildings: A novel hybrid CNN with a LSTM-AE based framework. Sensors. 2020 Mar 4;20(5):1399. doi: 10.3390/s20051399 32143371 PMC7085604

[27] Apadula F, Bassini A, Elli A, Scapin S. Relationships between meteorological variables and monthly electricity demand. Applied Energy. 2012 Oct 1;98:346–56. doi: 10.1016/j.apenergy.2012.03.053

[28] Zhu G, Peng S, Lao Y, Su Q, Sun Q. Short-term electricity consumption forecasting based on the EMD-fbprophet-LSTM method. Mathematical Problems in Engineering. 2021 Apr 12;2021:1–9. doi: 10.1155/2021/6613604

[29] Tian Y, Yu J, Zhao A. Predictive model of energy consumption for office building by using improved GWO-BP. Energy Reports. 2020 Nov 1;6:620–7. doi: 10.1016/j.egyr.2020.03.003

[30] Numata I, Cochrane MA, Souza CM Jr, Sales MH. Carbon emissions from deforestation and forest fragmentation in the Brazilian Amazon. Environmental Research Letters. 2011 Oct 10;6(4):044003. doi: 10.1088/1748-9326/6/4/044003

[31] Tso GK, Yau KK. Predicting electricity energy consumption: A comparison of regression analysis, decision tree and neural networks. Energy. 2007 Sep 1;32(9):1761–8. doi: 10.1016/j.energy.2006.11.010

[32] Chen T, Guestrin C. Xgboost: A scalable tree boosting system. InProceedings of the 22nd acm sigkdd international conference on knowledge discovery and data mining 2016 Aug 13 (pp. 785–794).

[33] Chen H, Tong Y, Wu L. Forecast of energy consumption based on FGM (1, 1) model. Mathematical Problems in Engineering. 2021 Feb 15;2021:1–1.

[34] Li K, Hu C, Liu G, Xue W. Building’s electricity consumption prediction using optimized artificial neural networks and principal component analysis. Energy and Buildings. 2015 Dec 1;108:106–13. doi: 10.1016/j.enbuild.2015.09.002

[35] Chinnaraji R, Ragupathy P. Accurate electricity consumption prediction using enhanced long short‐term memory. IET Communications. 2022 May;16(8):830–44. doi: 10.1049/cmu2.12384

[36] Karunathilake SL, Nagahamulla HR. Artificial neural networks for daily electricity demand prediction of Sri Lanka. In2017 Seventeenth International Conference on Advances in ICT for Emerging Regions (ICTer) 2017 Sep 6 (pp. 1–6). IEEE.

[37] Newsham GR, Birt BJ. Building-level occupancy data to improve ARIMA-based electricity use forecasts. InProceedings of the 2nd ACM workshop on embedded sensing systems for energy-efficiency in building 2010 Nov 2 (pp. 13–18).

[38] Shapi MK, Ramli NA, Awalin LJ. Energy consumption prediction by using machine learning for smart building: Case study in Malaysia. Developments in the Built Environment. 2021 Mar 1;5:100037. doi: 10.1016/j.dibe.2020.100037

